# The Genetic Regulation of Alternative Splicing in *Populus deltoides*

**DOI:** 10.3389/fpls.2020.00590

**Published:** 2020-06-05

**Authors:** Jerald D. Noble, Kelly M. Balmant, Christopher Dervinis, Gustavo de los Campos, Márcio F. R. Resende, Matias Kirst, William Brad Barbazuk

**Affiliations:** ^1^Plant Molecular and Cellular Biology Graduate Program, University of Florida, Gainesville, FL, United States; ^2^School of Forest Resources and Conservation, University of Florida, Gainesville, FL, United States; ^3^Department of Epidemiology and Biostatistics, Michigan State University, East Lansing, MI, United States; ^4^Department of Statistics and Probability, Michigan State University, East Lansing, MI, United States; ^5^Department of Horticultural Science, University of Florida, Gainesville, FL, United States; ^6^Genetics Institute, University of Florida, Gainesville, FL, United States; ^7^Department of Biology, University of Florida, Gainesville, FL, United States; ^8^Interdisciplinary Center for Biotechnology Research, University of Florida, Gainesville, FL, United States

**Keywords:** alternative splicing, population, isoform, poplar, splicing QTL, transcriptome, protein domain

## Abstract

Alternative splicing (AS) is a mechanism of regulation of the proteome via enabling the production of multiple mRNAs from a single gene. To date, the dynamics of AS and its effects on the protein sequences of individuals in a large and genetically unrelated population of trees have not been investigated. Here we describe the diversity of AS events within a previously genotyped population of 268 individuals of *Populus deltoides* and their putative downstream functional effects. Using a robust bioinformatics pipeline, the AS events and resulting transcript isoforms were discovered and quantified for each individual in the population. Analysis of the AS revealed that, as expected, most AS isoforms are conserved. However, we also identified a substantial collection of new, unannotated splice junctions and transcript isoforms. Heritability estimates for the expression of transcript isoforms showed that approximately half of the isoforms are heritable. The genetic regulators of these AS isoforms and splice junction usage were then identified using a genome-wide association analysis. The expression of AS isoforms was predominately *cis* regulated while splice junction usage was generally regulated in *trans*. Additionally, we identified 696 genes encoding alternatively spliced isoforms that changed putative protein domains relative to the longest protein coding isoform of the gene, and 859 genes exhibiting this same phenomenon relative to the most highly expressed isoform. Finally, we found that 748 genes gained or lost micro-RNA binding sites relative to the longest protein coding isoform of a given gene, while 940 gained or lost micro-RNA binding sites relative to the most highly expressed isoform. These results indicate that a significant fraction of AS events are genetically regulated and that this isoform usage can result in protein domain architecture changes.

## Introduction

The alternative splicing (AS) of pre-messenger RNA is a co-transcriptional process mediated by the spliceosome. This process can produce multiple messenger RNA (mRNA) isoforms from a single gene via the regulated usage of alternate splice sites during intron removal (Early et al., 1980; Saldi et al., 2016). AS was first identified in immunoglobulin μ (Early et al., 1980) and subsequent studies focused largely on single genes. However, advanced genome sequencing technologies have enabled rapid profiling of entire transcriptomes, and the large scale analysis of variation in AS both within species (Marquez et al., 2012; Thatcher et al., 2016) and between species (Barbosa-Morais et al., 2012; Chamala et al., 2015; Mei et al., 2017a).

Alternative splicing can regulate protein composition by changing the coding content between isoforms of the same gene. As a consequence, AS contributes to increased protein diversity and, ultimately, cellular and functional complexity, without increasing the size of a eukaryotic organism’s genome (Stamm et al., 2005). Alternative splicing events fall into five major categories: retained intron (RI), skipped exon (SE), alternative 5′ donor site (A5), alternative 3′ acceptor site (A3), and alternative 5′ or 3′ terminal exon (AE) (Barbazuk et al., 2008). In plants, RI is typically the most abundant event type and SE the least abundant (Marquez et al., 2012; Reddy et al., 2013; Chamala et al., 2015; Mei et al., 2017a, b). Furthermore, alternatively spliced isoforms containing premature stop codons are candidates for nonsense mediated mRNA decay (NMD). The NMD pathway targets mRNA transcripts containing stop codons upstream of a terminal exon junction for degradation. These unproductive transcripts serve to modulate the abundance of a gene’s protein product(s) without changing its expression (Schweingruber et al., 2013). AS also results in the production of gene isoforms that differ in protein domains, or in micro-RNA binding sites (Lewis et al., 2003; Thatcher et al., 2014). In plants, AS has been shown to impact the establishment of tissue identity and has been observed in response to environmental stimuli and pathogen infection (reviewed in Staiger and Brown, 2013; Szakonyi and Duque, 2018). The expression of alternatively spliced transcript isoforms is often tissue specific (Wang et al., 2008; Thatcher et al., 2014; Mei et al., 2017a). Advances in DNA sequencing technology and analysis methods have enabled the investigation of genome-wide patterns of AS in several plant species (reviewed in Szakonyi and Duque, 2018). Although the number of genome-level AS studies in plants has increased recently, they have been typically directed toward comparing treatments in a genotype of single species (Thatcher et al., 2016; Mei et al., 2017a), and, more recently, among species (Chamala et al., 2015; Mei et al., 2017b). In the meantime, relatively little is known about the dynamics of AS in a genetically diverse population of a species.

*Populus deltoides* (eastern cottonwood) is one of the most widely distributed tree species in North America and a keystone species in riparian ecosystems. There is also considerable commercial interest in its commercial cultivation because of its fast growth and potential use as a feedstock for the bioenergy industry. *P. deltoides* is used in poplar breeding to develop interspecific hybrid cultivars conferring traits for improved pulp and paper production, and improved feedstock quality for bioenergy production (Stanton et al., 2010). We recently reported the genotyping and genome-wide association study (GWAS) on a population of 391 *P. deltoides* unrelated individuals, in which significant associations were discovered for growth and wood composition traits (Fahrenkrog et al., 2017b). Here, we report the AS profile, the putative downstream effects of AS, and the genetic regulation of AS in differentiating xylem tissue of an association population of 268 *P. deltoides* individuals derived from the Fahrenkrog et al. (2017b) study. Large scale sampling of RNA across individuals was used to identify and characterize AS isoforms within each individual. We implemented a bioinformatics pipeline incorporating multiple transcript assembly programs and a reconciliation of their outputs. This enabled construction of a merged super-transcriptome representing all gene isoforms expressed in the population and their quantification, as well as the assessment of their genetic regulation.

## Materials and Methods

### Plant Material

The *P. deltoides* Bartr. ex Marsh (eastern cottonwood) association population analyzed in this study is composed of 343 individuals sampled from natural stands in 15 states in central, southern, and eastern United States. This population was vegetatively propagated and three biological replicates of each individual were grown in a greenhouse using a row-column design as previously described (Fahrenkrog et al., 2017a,b). After 16 weeks, stems were collected and differentiating xylem was separated from bark and phloem. Xylem was immediately flash-frozen in liquid nitrogen and stored at −80°C until lyophilization and RNA extraction.

### RNA Isolation

Lyophilized samples were ground in a Geno/Grinder tissue homogenizer (SPEX SamplePrep; Metuchen, NJ, United States). Total RNA was extracted by the CTAB-LiCl method (Chang et al., 1993). DNA contamination was removed using the TURBO^TM^ DNase kit (Invitrogen^TM^, Carlsbad, CA, United States). Total RNA concentration was quantified by the Qubit RNA Assay Kit with a Qubit 2.0 Fluorimeter (Life Technologies, Carlsbad, CA, United States). RNA purity and integrity were assessed using the Plant RNA Nano Kit and Bioanalyzer (Agilent Technologies, Santa Clara, CA, United States).

### Library Preparation and Sequencing

RNA-seq libraries were generated using NEBNext UltraTM Directional RNA Library Prep Kit for Illumina (NEB, Ipswich, MA, United States) following the manufacturer’s instructions. Briefly, the mRNA was purified from one μg of total RNA using poly-T oligo-attached magnetic beads. Next, mRNA was fragmented, reverse transcribed, and amplified (12 PCR cycles) using dual indexed primers provided in the NEBNext Multiplex Oligos for Illumina (Dual Index Primers Set 1, NEB #E7600L). Products were purified with the AMPure XP system (Beckman Coulter, Brea, CA, United States) and libraries were quantified using a Qubit 2.0 Fluorimeter. The libraries were assessed for quality and purity (Agilent Bioanalyzer 2100 system) before pooling in equimolar proportions. Ninety-six libraries were pooled and each pool was sequenced in paired-end mode (2 × 150 bp) using the HiSeq 3000 Sequencing System (Illumina, San Diego, CA, United States). Raw data, normalized and filtered gene expression data have been deposited in NCBI’s Gene Expression Omnibus (Edgar et al., 2002) and are accessible through GEO Series accession number GSE140232^1^.

### Sequencing Data Processing

On average, 24 Million PE reads were obtained/sample. RNA-seq FASTQ-files were pre-processed to remove low quality reads and to trim sequences based on quality scores with Trimmomatic v0.32 (Bolger et al., 2014) using the following parameters: TRAILING:3, SLIDINGWINDOW:4:20, MINLEN:20. The resulting quality-filtered reads were aligned to v3.0 of the *Populus trichocarpa* reference genome using STAR (Dobin et al., 2013) version 2.5.2b with parameters “–outFilterMismatchNmax 8 –sjdbOverhang 100 –outSAMtype BAM SortedByCoordinate –outSJfilterReads Unique –outSAMmultNmax 1 –outSAMattrRGline ID: < individual > _ < rep number > SM: < individual > _ < rep number > -outFilterType BySJout.” The SLIDINGWINDOW parameter (4:20) indicates that a sliding window of size 4 bases was used to scan the sequences 5′ to 3′. When the average quality falls below 20, the 3′ end of the read is truncated at that point. Average mapping rate after read filtering was 62% (vs. raw reads). Duplicate reads were removed with Picard v1.115^2^ using the parameter REMOVE_DUPLICATES = true and all other parameters as the default, keeping only unique alignments for further analysis.

### Transcriptome Assembly and Alternative Splicing Detection

Three transcript assembly platforms were used in order to maximize isoform detection: (i) Cufflinks version 2.2.1 (Roberts et al., 2011) with parameters “–library-type fr-firststrand –u -F 0.05 –max-intron-length 12000 –no-faux-reads -g”; (ii) StringTie version 1.3.3 (Pertea et al., 2015) with parameters “-f 0.05 -j 2 –rf”, and (iii) Trinity version 2.3.2 (Grabherr et al., 2011) in genome guided mode with parameters “–genome_guided_bam < input bam file > –genome_guided_max_intron 12000 –full_cleanup –SS_lib_type RF –min_contig_length 50.” The collection of Cufflinks and Stringtie isoforms detected for each sample were merged with Stringtie merge using parameters “-F 1 -f 0.05”. PASA version 2.0.2-r20151207 (Haas et al., 2003) was used to reconcile this merged assembly and the assembly from Trinity using parameters “-C –R -t < Trinity fasta output > –cufflinks_gtf < merged gtf file > -I 12000 –ALT_SPLICE –ALIGNER gmap,blat”. Additionally, the assemblies generated by PASA were filtered by requiring that (i) all splice junctions be supported by at least 2 reads, and (ii) retained introns be supported by a median read coverage of at least 2 (Python scripts stored in github.com/jdLikesPlants/poplar_AS). Requiring a minimum read support for RIs minimizes the possibility of incorrect identification of intron retention events from the sequencing of pre-mRNA (Marquez et al., 2012). Finally, the filtered PASA assemblies for each sample were merged with Cuffmerge (Cufflinks version 2.2.1) (Roberts et al., 2011) to generate a master transcriptome that represents all of the potential AS events and transcript isoforms for the population. The resulting assembly was then reformatted and annotated using gffcompare version 0.9.9c^3^. This transcriptome was subjected to a secondary expression-based filtering pipeline to remove artifacts generated during the merge. Cufflinks version 2.2.1 (Roberts et al., 2011) was used in quantification mode (parameters: –library-type fr-firststrand -G -u -F 0.05 –max-intron-length 12000) to measure the expression of the transcripts in the merged assembly in each sample. To minimize the presence of incorrectly assembled transcripts in the merged transcriptome assembly, each transcript was required to be expressed above FPKM (fragments per kilobase of exon model per million reads mapped) 3 in at least two of three biological replicates of a given individual, and in at least 3 individuals in the population. This final merged and filtered transcriptome was used in all downstream analyses. The GTF file describing the merged and filtered transcriptome is available on Figshare: https://figshare.com/articles/Poplar_Isoform_Expression_matrix_zip/12091530

An overview of this computational pipeline is depicted in Supplementary Figure 1. Additionally, any individual that did not have at least 15 million reads generated during sequencing in at least two of three replicates, as well as individuals for which only one replicate was sequenced were removed from analysis, resulting in a final set of 268 individuals.

### AS Classification and Transcript Quantification

Five types of AS events were considered in this study: alternative 3′ acceptor (A3), alternative 5′ donor (A5), intron retention (RI), exon skipping (ES), and alternative 5′ or 3′ terminal exon (AE). PASA (Haas et al., 2003) was used to profile the types and abundance of AS events in the transcriptome. Cufflinks (Roberts et al., 2011) was used with parameters “–library-type fr-firststrand –u -F 0.05 –max-intron-length 12000 -G” to quantify transcript abundance. Presence/absence variation of alternative splicing was analyzed at the isoform expression level where a given isoform was considered present in an individual if it was expressed at or above an FPKM of 3 in at least two biological replicates. A matrix of isoform expression values for each sample in the population is presented in Figshare^4^.

### SNP Liftover From *P. trichocarpa* Hybrid Genome to *P. trichocarpa* V3 Coordinates

A collection of 358809 high quality single nucleotide polymorphisms (SNPs) previously identified for this population by Fahrenkrog et al. (2017a) using a hybrid *Populus* genome assembly were converted to locations in the *P. trichocarpa* v3.0 reference genome assembly using a modified liftover pipeline^5^. The liftover pipeline uses a series of scripts from the Kent tools collection^6^, CrossMap (Zhao et al., 2014), BEDOPS (Neph et al., 2012), and a custom python script to consolidate the converted coordinates. This pipeline extracts the sequence flanking a SNP from the genome where it was identified and aligns the sequence to the genome one wishes to convert the SNP coordinates. A stepwise document of the liftover pipeline can be found at github.com/jdLikesPlants/poplar_AS/liftover. In total, 313036 of the 358809 SNPs were converted to *P. trichocarpa* v3 reference genome coordinates. The SNP set was then filtered to remove SNPs that departed from Hardy-Weinberg equilibrium by removing those with a p-value smaller than 0.01 (chi square test). Additionally SNPs with a minor allele frequency below 0.05, a mean sequencing depth below 8, genotype quality below 20, and missing data above 75% were removed. The resulting 68,885 SNPs were used in the analysis that followed.

### Gene and Isoform Heritability Measures

We used a standard linear mixed model to estimate the variance for each of the random effects, including genotype ($\sigma_{g}^{2}$), row ($\sigma_{r}^{2}$), column ($\sigma_{c}^{2}$), and errors ($\sigma_{\varepsilon }^{2}$). Separate models were fitted to each one of the genes and isoforms using the lmer function of the lme4 R package (Bates et al., 2015). The response was transcript abundance (FPKM), and the model included an intercept, the fixed effect of the experiment, and the random effect of the genotype, row, and column within the experiment. From the variances’ estimates we estimated gene- and isoform-specific heritability using:

$$H^{2}=\frac{\sigma_{g}^{2}}{\sigma_{g}^{2}+\sigma_{r}^{2}+\sigma_{c}^{2}+\sigma_{\varepsilon }^{2}}$$

Subsequently, we conducted 10,000 permutations of the data, from where we approximated the distribution of the gene and isoform–specific heritabilities under the null hypothesis, $\sigma_{g}^{2}=0$. This was done by permuting the genotype ID while maintaining the rest of the data structure unchanged. Following this approach, a total of 20,737 isoforms with heritability greater than the 99th percentile estimates from the permutation test (*p*-value < 0.01) were kept for subsequent analysis.

### Gain/Loss of Protein Domains Between Alternatively Spliced Gene Isoforms

The switching of protein domains was identified with the Python script^7^ “domain_gain_loss_longest.py” using the “.indiv_splice_labels_and_coords” file generated by PASA (Haas et al., 2003) and the coordinates of the protein domains coded by the isoforms generated by Pfam-scan (Mistry et al., 2007) converted to genomic coordinates. The protein domains of the longest isoform and major (most highly expressed) isoform of a gene were compared to all other isoforms of the gene in two separate analyses. If the alternative isoform(s) contained a domain that the longest/major isoform lacked, it was considered to have gained a domain. In contrast, a loss was determined if the alternative isoform(s) lacked a domain detected in the longest/major isoform. The coordinates at which the gain or loss of protein domains occurred was then cross referenced to the coordinates of the alternative splicing events associated with the isoforms to identify AS events affecting the gain or loss of protein domains between isoforms.

### Gain and Loss of Micro RNA Binding Sites in Alternatively Spliced Isoforms

The sequences of all transcripts generated as described above in were used as inputs to psRNATarget (Dai et al., 2018) to identify putative miRNA target sites. Default parameters for scoring and filtering were used. The locations of the splice sites were converted to genomic loci with Python script “get_miRNA_binding_genomic_coords.py”. A similar analysis to the gain/loss of protein domains was done to identify which miRNA binding sites were gained or lost as a result of alternative splicing. The binding sites of the longest protein coding isoform or major (most highly expressed) isoform of a gene were compared to all other isoforms of a gene in two separate analyses.

### Identification of Isoform Expression Quantitative Trait Loci (iso-eQTL) and Splicing Quantitative Trait Loci (sQTL) Regulating Transcript Isoform Expression

iso-eQTL and sQTL were identified using Matrix eQTL (Shabalin, 2012). Loci associated with an iso-eQTL or sQTL and located within 1 Mbp of the transcript position we considered to be *cis*- regulators. The isoform expression levels generated by Cufflinks (Roberts et al., 2011) were averaged for all of the replicates for each individual and used as inputs to identify iso-eQTL. Splice junction usage estimates were generated by dividing the number of reads spanning a gene’s splice junctions divided by the number of reads mapping to a gene. These estimates were averaged across all biological replicates of each individual before being used as inputs to identify sQTL. For a splice junction to be considered for analysis it was required to be associated with an AS event detected by PASA (Haas et al., 2003) and for the estimate of its usage to be above 0 in at least 5% of the individuals in the population. The number of reads mapping to a gene were generated via htseq-count (Anders et al., 2015) and the number of reads spanning a splice junction was generated with a custom awk script available in the github repository for this project.

## Results

### Discovery of Alternative Splicing Events and Novel Isoforms in *Populus deltoides* Differentiating Xylem

**FIGURE 1:**
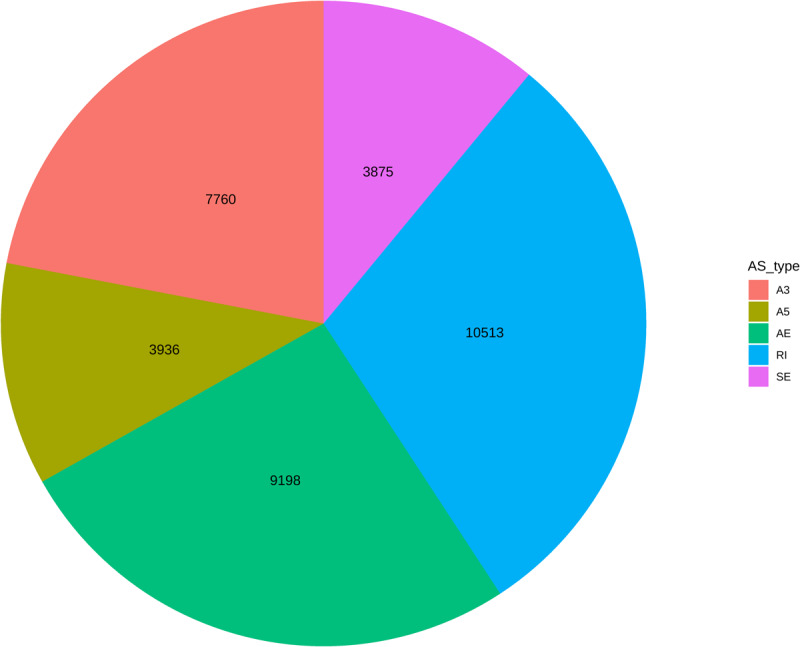
Summary of the abundance of alternative splicing event types. Colors associated with each event type are in the color legend. Abbreviations in legend are defined as: A3, alternative 3′ acceptor sire; A5, alternative 5′ donor site; AE, alternative terminal exon; RI, retained intron; SE, skipped exon.

To assess the complexity of AS in the *P. deltoides* differentiating xylem, the transcriptome assemblies derived from each biological replicate of 268 individuals of the population were merged into a single reference representing all transcribed genes and transcript isoforms. Using this reference transcriptome, we investigated AS types, quantified their abundance, and the relation between the number of isoforms a gene expresses and the genomic features of that gene. In total, 35,282 AS events were detected for 12,259 genes. The number of genes found to undergo AS in this study was similar in number (11,232) to those seen in a previous study of *P. trichocarpa* in (Bao et al., 2014) and maize (14,321) that were discovered using a similar in transcriptome assembly pipeline and filtering (Mei et al., 2017b). The GTF file describing these AS isoforms is available at FigShare^8^. RI was the most common AS type (10513), while SE was found to be the least common in the population (3,875 events) (Figure 1). For the 12,259 genes that exhibited AS, the number of splice junctions ranged from 2 to 61, with a mean of 8.85 (median 7). Next, we investigated the association between the number of isoforms encoded by a gene and other gene characteristics, such as gene length, number of exons, and gene expression. Significant positive correlations were identified between the length of genes and the number of encoded isoforms (*r*^2^ = 0.44, *p*-value < 1e-16), and between the number of exons and number of isoforms (*r*^2^ = 0.46; *p*-value < 1e-16). A highly positive correlation was also detected between the median expression of a gene and the number of isoforms (*r*^2^ = 0.66; *p*-value < 1e-16). These results suggest that higher transcript abundance, longer genes, and the number of exons provide more opportunities for the occurrence of AS events.

Next, we compared the merged transcriptome to the *P. trichocarpa* v3.0 reference genome annotation. Based on the merged transcriptome, 22,429 genes were identified as being expressed in the differentiating xylem of *P. deltoides*. A total of 48,796 transcript isoforms were observed for these genes, of which 10,170 originate from non-AS genes and 38,626 from AS genes. Note that A3 and A5 events yield at least two isoforms for each event. Of the 48,796 isoforms that occur in the population, 14,214 were previously described in the *P. trichocarpa* v3 reference genome annotation, while 38,582 represent novel isoforms discovered by the transcriptome assembly pipeline. The number of transcript isoforms per annotated gene ranged from 1 to 20 with a mean of 2.33 (median: 2). Among the 22,429 genes expressed in the population, 1709 represent transcriptional units not previously described in the *P. trichocarpa v3.0* reference annotation. Novel transcriptional units were found on average in 61 individuals (median 24) of the population. We performed a BLAST search of these novel transcriptional units against the Arabidopsis gene database and found 136 annotated homologs. 100 of these transcripts encoded open reading frames and protein family (Pfam) domains were identified for 57 (Supplementary Table 1). Among the unannotated transcripts, 116 encoded two or more isoforms (average:2.33 isoforms per gene, median: 2). Their transcript abundance in the population was relatively high, with a mean expression level of 11.55 (median: 2.97).

### Genetic Heritability of Isoform Expression

**FIGURE 2:**
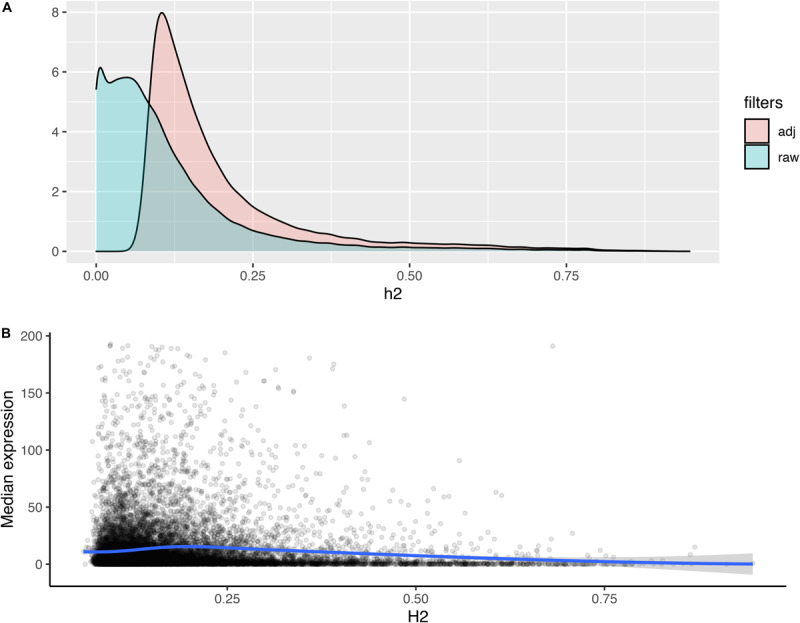
Distribution of heritability estimates and its relation to isoform expression. **(A)** Stacked bar plot of the distributions of heritability estimates for all isoforms (raw) and the subset of isoforms with heritability greater than expected by chance (adj; permutation test, *p*-value < 0.01). **(B)** Loess plot depicting the correlation between the median expression of an isoform (y) and the heritability estimate for an isoform (x). Blue line represents *r*_s_ = –0.14. Density plot of the distributions of heritability estimates for all isoforms (raw) and the subset of isoforms with heritability greater than expected by chance (adj; permutation test, *p*-value < 0.01). **(B)** Loess plot depicting the correlation between the median expression of an isoform (y) and the heritability estimate for an isoform (x).

The broad-sense heritability (*H*^2^) of gene expression provides an estimate of the transcriptional variance observed in a population that is determined by genetic factors, relative to the total variance. We calculated *H*^2^ for all expressed isoforms, in order to assess the extent by which genetic variance contributes to isoform expression variance (Figure 2A). Among the 48,796 transcript isoforms detected in the population, 20,737 isoforms had *H*^2^ higher than expected by chance (*p*-value < 0.01; permutation testing), including 1153 isoforms with heritability higher than 0.5 (Figure 2A). In general, isoforms that were more highly expressed exhibited significantly lower heritability estimates. More specifically, a negative correlation (*r*_s_ = −0.09; *p*-value 1.85e-29) was detected between *H*^2^ and the median expression of an isoform in the population (Figure 2B).

### Transcript Isoform Presence/Absence and Frequency Variation in the Population

**FIGURE 3:**
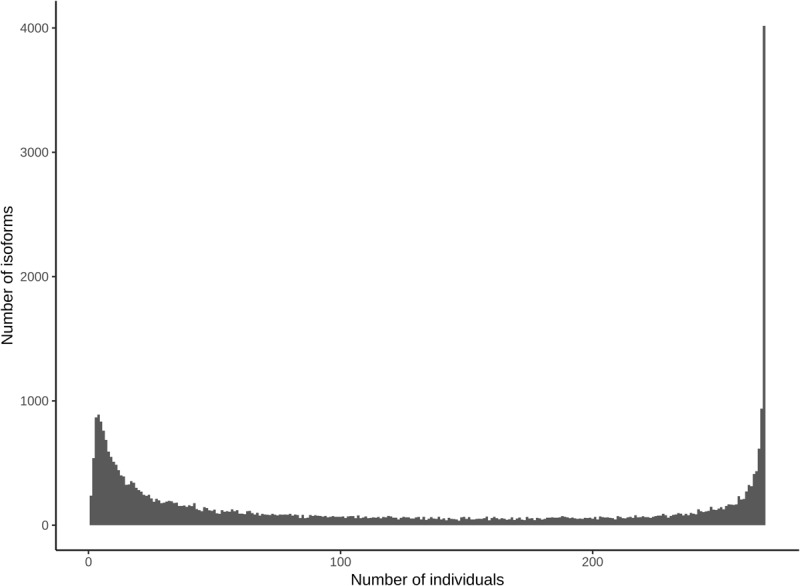
Presence/absence variation of isoforms in the population. *X*-axis denotes the number of individuals an isoform was present in and the *y*-axis denotes the sum of isoforms present in that number of individuals.

Alternative transcript isoforms may provide distinct molecular and cellular functions, and are often tissue specific (Wang et al., 2008). To begin uncovering the role of the multiple isoforms for genes expressed in differentiating xylem, we initially evaluated presence and absence variation (PAV) of AS in the *P. deltoides* population. A given isoform was declared present if it was expressed at or above an FPKM of 3 in at least two of the biological replicates of a given individual. Using this approach, we found that most of the observed isoforms are detected throughout all individuals in the population (Figure 3). Thus, isoform presence in differentiating xylem does not appear to be highly variable among individuals in this population.

**FIGURE 4:**
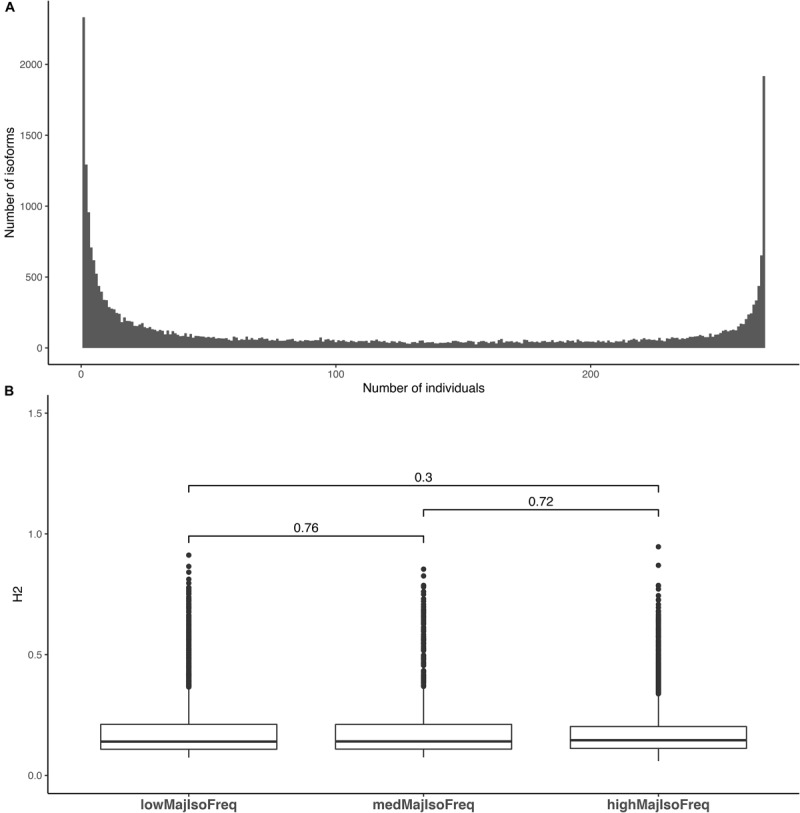
Distribution of major isoform frequencies and heritability distributions within major isoform frequency classes. **(A)** The number of individuals in which an isoform is the most expressed isoform. The *y*-axis for this figure corresponds to the number of isoforms with a major isoform frequency at the *x*-axis location. **(B)** Heritability distribution of isoforms with low (<20% of the population), medium (40–60% of the population) and high (90%) major isoform frequencies. Bars above the boxplots denote the comparisons made using a Mann–Whitney test and the values above the bars denote the *p*-value acquired from the test.

Although most isoforms were detected across most of the population, there may be quantitative variation in the level of transcription detected in each one of them. To address the question whether for each gene a specific isoform is predominantly expressed in the population, we calculated the major isoform frequency. For this, we estimated the number of individuals in which each isoform was the most highly expressed, relative to the other isoforms for each gene. The distribution of major isoform frequencies was bimodal. While the most expressed isoform was often the same in all individuals of the population, in some instances the most expressed isoform was detected in only few individuals (Figure 4A). We also explored if there was a relationship between the major isoform frequency and its heritability, but observed no significant difference in heritability estimates among isoforms that occur with high (>90%), medium (40–60%), and low (<20%) major isoform frequencies (Figure 4B). Thus, the most highly expressed isoform of a gene is not necessarily the most heritable in the population. Data representing the number of individuals that an isoform was present in, and the median isoform fraction of that isoform are presented in Supplementary Table 2; a matrix of isoform expression values for each sample in the population is available from Figshare^9^.

### Gain and Loss of Protein Domains in Alternatively Spliced Isoforms

To provide insight into how AS affects the coding sequence of a translated mRNA, we examined AS events to determine which resulted in the gain or loss of protein domains (see section Materials and Methods). Because AS changes the nucleotide content of an mRNA, it follows that AS can also change the function of the resulting protein by modifying its domain content (Thatcher et al., 2014; Climente-González et al., 2017).

We first classified protein domain gains or losses by comparing the isoforms of each AS gene to the longest protein coding isoform encoded by that gene. Following the identification of which protein domains were gained or lost, we determined which AS events were implicated in changes to the domain architecture. A total of 696 genes were found to have AS isoforms that gained or lost domains relative to the longest protein coding isoform of the gene. Next, we performed a similar analysis, but using the most highly expressed isoform as a reference (instead of the longest) to which the other isoforms were compared to. This resulted in the detection of 859 genes in which isoforms gained or lost domains relative to the most expressed isoform. In total, 498 genes overlapped between the genes exhibiting domain gain/loss between isoforms using the longest protein coding isoform or the most highly expressed isoform as a reference and 45.4% of these gene’s isoforms with domain architecture affected by AS were significantly heritable. The domains affected in these three gene sets are included in Supplementary Table 3.

Enrichment analysis was performed with PopGenIE (Sjödin et al., 2009) to assess if there are categories of gene ontology (GO) terms, Pfam domains, or KEGG IDs that are overrepresented among the genes that underwent changes in domain architecture. Such enrichment would suggest that a given category of genes is more amenable to changes in protein domain architecture.

The only GO term enriched in the 696 genes with domain gain/loss relative to the longest protein coding isoform of a gene was “nucleic acid binding.” Pfam enrichment was detected for “RNA recognition motif” (PF00076) and the most enriched KEGG term was “small nuclear ribonucleoprotein D2.” GO terms enriched for the 859 genes with domain gain/loss relative to the most highly expressed isoform of a gene were “intracellular transport” and “protein binding.” The top Pfam enrichments were “Tetratricopeptide repeat” (PF00515) and “RNA recognition motif” (PF00076). The 498 genes overlapping between the genes with domain gain/loss relative to the most highly expressed isoform and the longest isoform of a gene were significantly enriched for the GO term “nucleic acid binding,” while the most enriched Pfam ID was PF00076 “RNA recognition motif (a.k.a. RRM, RBD, or RNP domain),” and most enriched KEGG IDs were “small nuclear ribonucleoprotein D2” and “5′-methylthioadenosine nucleosidase.” The complete results for all three gene sets are provided in Supplementary Table 4.

Because the term “RNA recognition motif” was very highly enriched for every gene set, functional annotation was done using the Arabidopsis orthologs for the Poplar genes containing this motif with DAVID (Huang et al., 2009) which showed these genes to be enriched in processes involved in alternative splicing (Supplementary Table 5).

### Gain and Loss of Micro RNA Binding Sites in Alternatively Spliced Isoforms

MicroRNAs (miRNA) can contribute to RNA silencing and post-transcriptional regulation through the action of small non-coding RNA molecules that bind to specific sites. Thus, the loss or gain of such sites could impact gene function. We assessed the consequences of AS on miRNA binding sites using an approach similar to that described above for the gain or loss of protein domains. A total of 748 genes had predicted miRNA binding sites that were modified relative to the longest protein coding isoform, while 940 genes showed changes occurring relative to the most highly expressed isoform. There were 698 genes common to the two gene sets. The miRNA binding sites affected in these three gene sets are provided in Supplementary Table 6.

Functional categories were detected for these 698 genes with PopGenIE (Sjödin et al., 2009). Enrichment results are provided in Supplementary Table 7. The most highly enriched GO terms were “phosphoenolpyruvate carboxykinase activity” and “nucleic acid binding,” while the most common Pfam domain was “RNA recognition motif” (PF00076). Functional enrichment was done with DAVID (Huang et al., 2009) with the Arabidopsis orthologs of the RNA Recognition Motif containing genes from the set of 698 genes. Results indicate that this gene set is enriched in processes involved in alternative splicing (Supplementary Table 8).

When comparing the gain/loss of miRNA binding sites between AS isoforms relative to the longest protein coding isoform of a gene, 7 genes had at least one isoform that gained a binding site and at least one that lost a binding site, 536 genes had isoforms that lost binding sites, and 205 genes had isoforms that gained binding sites. This trend was reversed when comparing the gain/loss of miRNA binding sites relative to the major isoform of a gene. When comparing to the major isoform of a gene, 9 genes had at least one isoform that gained a binding site and one that lost a binding site, 277 genes had isoforms that lost binding sites, and 654 genes had isoforms that gained binding sites.

### Identification of iso-eQTL and Splicing QTL Regulating Transcript Isoform Expression and Splice Junction Usage

**FIGURE 5:**
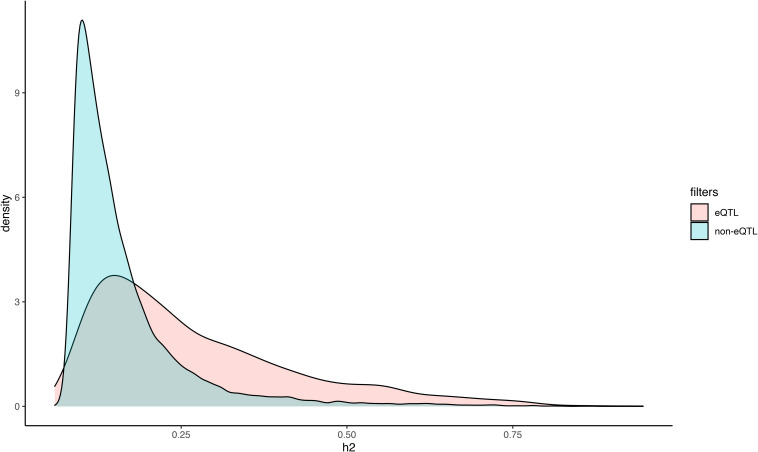
Density plot of the distribution of heritability estimates for transcript isoforms with, and without, significant iso-eQTL. Isoforms were partitioned based on whether or not they had significant iso-eQTL and the distribution of the heritability estimates were plotted.

The genetic regulation of gene expression has been extensively characterized in plants, but these studies have generally considered all isoforms combined as a quantitative representation of the transcription of each gene in an individual. Considering that we generated RNA-seq data and isoform quantification for a previously genotyped population, we attempted to use this information to map the genetic loci that control individual isoform expression. iso-eQTL were identified for all heritable isoforms encoded by alternatively spliced genes using Matrix eQTL (Shabalin, 2012) (see section “Materials and Methods”). We detected a total of 11,204 iso-eQTL associations at a 5% empirical FDR. These represent significant associations between the expression of 2,435 isoforms and 6,236 SNPs. The iso-eQTL were classified as *cis* if the regulatory SNP was located up to 1 Mb upstream of the transcription start site, or 1 Mb downstream of the transcription stop site, of the isoform it was putatively regulating. Alternatively, iso-eQTL were classified as *trans*. According to this classification, 3,572 were *cis* regulating 1,924 unique isoforms and 3,119 were *trans* regulating 903 unique isoforms. We found that 2,417 of the *cis* iso-eQTL were located within exons, 529 within introns, and 626 within intergenic regions, based on the transcriptome annotation of this population. Additionally, 1,910 of the *trans* iso-eQTL were located within exons, 336 within introns, and 871 within intergenic regions. Finally, we observed that isoforms with iso-eQTL associations showed significantly higher heritability estimates (Mann–Whitney *U* test; *p*-value 9.86e-217) than heritable isoforms without iso-eQTL (Figure 5).

To identify putative splicing QTL (sQTL), Matrix eQTL (Shabalin, 2012) was utilized using the percentage of reads mapping to a gene’s splice junctions associated with AS events (see section “Materials and Methods”). In total, 911 unique *cis*- and 7097 unique *trans*-sQTL were identified at a 5% empirical FDR regulating 3219 splice junctions contained in 2569 genes. Genes regulated by sQTL, and genes harboring *cis*-sQTL, encoded significantly more heritable isoforms per gene than genes without sQTL associations (Table 1).

**TABLE 1:** Summary of the number of heritable isoforms per gene for genes regulated by sQTL or harboring sQTL (regulator genes).

**Class**	**Mean reg**	**Mean non-reg**	**pvalue**
*Cis* regulated	2.45	1.14	2.72E-62
*Trans* regulated	1.61	1.07	7.50E-70
*Cis* regulator	1.81	1.16	1.21E-13
*Trans* regulator	1.20	1.18	0.998

*The mean number of heritable isoforms per gene for *cis* and *trans* regulated (or regulator) genes is provided along with the mean number of heritable isoforms for non-regulated (or regulator) genes. The “mean reg” column denotes the mean number of heritable isoforms pre regulated gene for the corresponding class. The “pvalue” column denotes the *p*-value derived from a Mann–Whitney *U* test comparing the number of heritable isoforms per gene between cis, and trans, regulated (or regulator) and non-regulated (or non-regulator) sQTL genes.*

**FIGURE 6:**
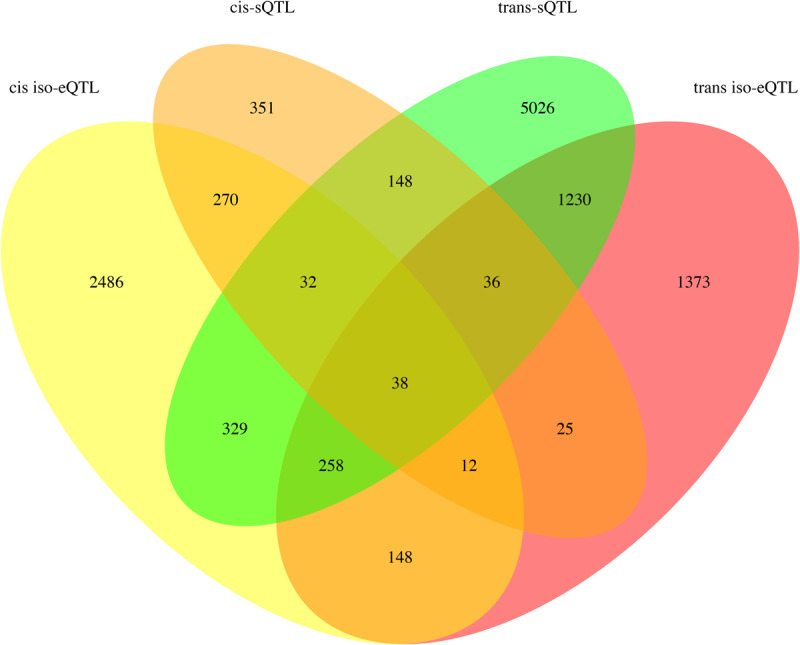
Venn diagram depicting the sharing of SNPs between SNP sets involved in *cis* and *trans* regulation of iso-eQTL and sQTL.

Loci regulating isoform expression or splice junction usage were distinct from each another (Figure 6). The AS events regulated by sQTL are provided in Supplementary Table 9. A total of 488 RI, 309 A5, 516 A3, and 120 SE junctions were *cis* regulated and 413 RI, 187 A5, 385 A3, and 95 SE junctions were *trans* regulated. Note that a given splice junction may be associated with more than one AS event. For instance, a junction with an alternative 5′ acceptor in one gene isoform may also have an intron retained at this junction in a different isoform.

## Discussion

In this study we investigated the genetic regulation of alternative splicing and its functional consequences in a sample of genetically unrelated individuals of the non-domesticated tree species *P. deltoides*. We found that AS is self-regulating via the gain and loss of protein domains and via the gain and loss of miRNA binding sites in RNA recognition motif containing genes. We also found that contrary to the genetic regulation of transcript expression, the genetic regulation of splice junction usage is *trans* rather than *cis* regulated. This study increases the understanding of population scale alternative splicing in the *Populus* genus previously found by Bao et al. (2013). Additionally, it provides perspective into the heritability of alternative splicing by the proportion of isoform expression variance explained by the genetic variance in the samples under investigation. To our knowledge, this is the first study to investigate the genetic regulation of alternative splicing and provide a comprehensive overview of its downstream consequences in a forest tree species.

By leveraging multiple assembly programs, followed by stringent splice junction read support filtering, we generated a robust transcriptome representing all observed isoforms expressed in differentiating xylem, and calculated the extent of their genetic regulation. The downstream consequences of these splicing events were also investigated using the predicted *in silico* translated peptide sequences generated. Finally, loci that contribute to the expression of transcript isoforms and splice junction usage were detected.

An initial analysis of transcriptome showed that 1,709 of the expressed genes identified represent potentially novel, unannotated open reads frames. The discovery of new transcriptional units not present in a reference genome is common when performing transcriptome assemblies (Schliebner et al., 2014). The large proportion of novel isoforms within annotated genes (∼63%) compared to isoforms in the *P. trichocarpa* v3.0 reference annotation may be attributable to the use of RNA-seq data from *P. deltoides* rather than *P. trichocarpa*. Mis-assembly and/or chimeric reads could not explain the existence of the “novel genes” as the depth of coverage was generally high and as evidenced by the fact these newly identified transcriptional units were typically found in average several individuals of the population. Moreover, many of these new transcriptional units are potentially tissue-specific and expressed only in xylem.

While AS has been extensively characterized across different species and tissues (Chamala et al., 2015; Mei et al., 2017a, b), there is relatively limited understanding of the extent by which the variance of individual isoform expression is genetically regulated and, therefore, heritable. By characterizing isoform expression in a *P. deltoides* population we were able to estimate the heritability of all isoforms for each gene and uncover the extent by which its variance is attributable to genetic variation. The quantitative genetic analysis of transcript isoform abundance showed that the expression of a large fraction of them (43%) is heritable. While large, this proportion is contrastingly smaller relative to the gene-level heritability (i.e., combining all isoforms representing a gene into one transcriptional unit) reported for this population (69%, Balmant et al., 2020). Lower heritability at the isoform level suggests that there may be more limited genetic regulation (i.e., non-genetic factors, such as the environment, contribute more extensively to the variation observed) or limited genetic variation in the control of expression of a certain fraction of the isoforms that represent a gene. In an extreme scenario, a single isoform would be heritably expressed for each individual gene, suggesting that the expression detected in the remaining isoforms is the consequence of non-genetic regulation and/or technical or molecular noise. We discarded this hypothesis by quantifying the number of isoforms that are heritable for each individual gene that contained multiple isoforms and observed that in most cases two or more isoforms have a heritability significantly higher than 0. We also measured the relationship between expression of isoforms and their heritability, considering that weakly transcribed isoforms may be unreliably quantified (therefore resulting in lower heritabilities) or the consequence of regulation driven by non-genetic factors. However, we detected very limited correlation between the level of expression of isoforms and their heritability – that is, more highly expressed isoforms are not necessarily the most heritable.

While the heritability provides an estimate of the extent of variation attributable to a genetic component, the genotypic data available for this population created the opportunity to also identify iso-eQTL and sQTL. Based on this analysis, the majority of iso-eQTL were designated as *cis* based on their proximity to the transcript they were regulating while the majority of sQTL were *trans*. In eQTL studies it is often found that a large fraction of genes are regulated in *cis* (Ranjan et al., 2016; Mähler et al., 2017) and for eQTL and sQTL to be independent of each other (Li et al., 2016). Our findings were similar in that the majority of iso-eQTL were *cis* and iso-eQTL were distinct from sQTL. In fact, only 95 genes had their different isoforms distinctly regulated in both *cis* and *trans*. The majority of sQTL however, were *trans*. Previous sQTL studies in humans focused their analysis only to variants that were proximal, or in *cis*, to AS genes (Li et al., 2016; Takata et al., 2017). Our findings indicate that variants regulating splice junction usage that are distant from the AS genes should be considered when investigating sQTL.

Isoform expression has been often shown to be tissue specific – that is, a single isoform of an AS gene is predominately expressed in a given tissue (Wang et al., 2008; Thatcher et al., 2014). Tissue specific expression of alternatively spliced isoforms plays roles in plant growth, development, and the establishment of tissue identity (Staiger and Brown, 2013). The expression of major isoforms, the most highly expressed isoform of a given gene, has been shown to mostly conserved among individuals in human tissues such that the same isoform is the major isoform in the majority of the population studied (Melé et al., 2015). While this study evaluated isoform expression exclusively in differentiating xylem, the analysis of a large population allowed us to assess if specific isoforms are predominant or if multiple, alternative isoforms may be the most expressed in different individuals of the population. We observed that the former case is most common – 69% of transcript isoforms were predominantly expressed in over 90% of the population. Therefore, most genes have a single isoform that constitutes the majority of transcript expression. This result agrees with the findings in Melé et al. (2015) where inter-individual isoform expression variation was found to be relatively low. This result was also similar to the analysis by Bao et al. (2013) which investigated the conservation of alternative splicing at the splice junction level across a small collection of 20 individuals of *Populus trichocarpa*. Further work that investigates isoform frequencies using RNA-seq libraries from multiple plant tissues in a diverse population will determine if other isoforms are the most highly expressed in different tissues.

Alternative splicing increases the complexity of the proteome without changes to the genome by producing multiple mRNA species from a single gene. Changing the sequence of an mRNA can change the sequence of the peptide translated from it. Previous AS studies in plants have uncovered AS events affecting specific protein domains (Thatcher et al., 2014, 2016). However, to the best of our knowledge, a comprehensive overview of protein domain architecture modified by AS events in plants has not been reported. Here we developed a pipeline to reproducibly identify AS events modifying protein domains and showed that a reasonably high number of genes (∼500) are affected by these changes. Enrichment analysis for the genes with modified domain architecture revealed that only genes involved in RNA binding (GO term: nucleic acid binding and PFAM: RNA recognition motif) were enriched among the set of genes affected by this process. Functional enrichment clustering of the genes in this category with DAVID (Huang et al., 2009) showed them to be involved in AS; thus AS is a self-regulating mechanism by altering the gain/loss of the protein domains of the splicing machinery.

Similarly, miRNA binding sites are also affected as a result of AS. The gain or loss of miRNA binding sites as a result of AS between tissues and developmental stages in maize was reported previously (Thatcher et al., 2014). Our analysis revealed that the most highly expressed isoform of a gene more often lacked a miRNA binding site that lower expressed isoforms of the gene contained. This finding may be the result of post-transcriptional down-regulation of minor isoforms of genes by the miRNAs targeting minor isoforms. Enrichment for genes with modified miRNA binding sites from AS also were enriched for the Pfam domain RNA recognition motif. Functional enrichment clustering with DAVID (Huang et al., 2009) showed these genes to be involved in AS, therefore AS also self-regulates via the change in miRNA binding sites between gene isoforms. The only highly enriched GO term for genes with miRNA binding sites affected by AS was “Phosphoenolpyruvate carboxykinase activity.” There are four genes annotated as phosphoenolpyruvate carboxykinase (PCK) in the genes with miRNA binding sites affected by AS. PCK was found to be upregulated in *Populus* hybrids exposed to high levels of nitrogen whose xylem architecture was remodeled as a result of this treatment (Plavcova et al., 2013). These four genes were highly expressed in the population (median FPKM: 50.44, 31, 30.2, 20.67) and all had significantly heritable isoforms. Further investigation of these transcripts and their miRNA binding sites may reveal effects on xylem architecture and nitrogen use.

## Conclusion

The analyses in this study provides a description of alternative splicing in a large sample of unrelated individuals of *P. deltoides*, which is commonly used in *Populus* breeding programs. A previous study in *Populus* investigated AS in a population of 20 genotypes (Bao et al., 2013). Here we expand this by profiling AS at the population transcriptome level, identifying putative genetic regulators of AS, and identifying the AS events that affect the gain or loss of protein domains and miRNA binding sites. The genes with genetically regulated AS and protein domain changes resulting from AS may be incorporated into genomic selection models to improve their robustness via serving as markers for selection. These genes may also be further analyzed for their potential role on phenotypic traits.

## Data Availability Statement

The datasets generated for this study can be found in the Gene expression omnibus https://www.ncbi.nlm.nih.gov/geo/query/acc.cgi?acc=GSE140232, A GTF file describing the merged and filtered transcriptome and a matrix of isoform expression is available from Figshare at https://figshare.com/articles/Poplar_Isoform_Expression_matrix_zip/12091530.

## Author Contributions

JN, MK, and WB designed the experiments. JN, KB, and CD performed RNA-seq experiments. JN performed isoform assemblies and related bioinformatics analysis. JN, KB, and GC performed the statistical analyses. MR assisted in heritability estimations. JN, MK, and WB analyzed data. JN, KM, MK, and WB wrote the manuscript.

## Conflict of Interest

The authors declare that the research was conducted in the absence of any commercial or financial relationships that could be construed as a potential conflict of interest.

## Footnotes

1 https://www.ncbi.nlm.nih.gov/geo/query/acc.cgi?acc=GSE140232

2 https://broadinstitute.github.io/picard/

3 https://github.com/gpertea/gffread

4 https://figshare.com/articles/Poplar_Isoform_Expression_matrix_zip/12091530

5 http://genomewiki.ucsc.edu/index.php/Minimal_Steps_For_LiftOver

6 https://users.soe.ucsc.edu/~kent/

7 https://github.com/jfLikesPlants/poplar_AS/tree/master/domain_gain_loss

8 https://figshare.com/articles/Poplar_Isoform_Expression_matrix_zip/12091530

9 https://figshare.com/articles/Poplar_Isoform_Expression_matrix_zip/12091530

## Supplementary Material

FIGURE S1 A flowchart depicting the transcriptome assembly and filtering pipeline.

TABLE S1 Pfam domains identified for novel isoforms identified during transcriptome assembly.

TABLE S2 Protein domains affected by AS relative to the longest and most expressed isoforms. Additionally, those domains overlapping between these two sets.

TABLE S3 Popgenie enrichment results for the genes with protein domains affected by AS.

TABLE S4 DAVID enrichment clustering for Arabidopsis orthologs of Poplar genes with RNA recognition motif domains affected by AS.

TABLE S5 miRNA binding sites affected by AS relative to the longest and most expressed isoforms. Additionally, those domains overlapping between these two sets.

TABLE S6 Popgenie enrichment results for the genes with miRNA binding sites affected by AS.

TABLE S7 DAVID enrichment clustering for Arabidopsis orthologs of Poplar genes with RNA recognition motif domains that had miRNA binding site gain/loss affected by AS.

TABLE S8 AS events regulated by sQTL.
