# Supplementary figures and images for: The Genetic Regulation of Alternative Splicing in Populus deltoides
Source: Front Plant Sci. 2020 Jun 5;11:590. doi: 10.3389/fpls.2020.00590 (PMC7291814; doi:10.3389/fpls.2020.00590)

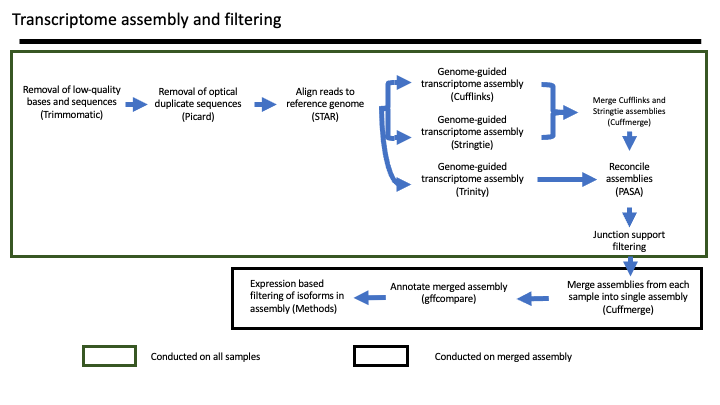

Supplement: FIGURE S1 — A flowchart depicting the transcriptome assembly and filtering pipeline. [file Image_1.TIFF]
